# Modelling the Health Policy Process: One Size Fits All or Horses for Courses?

**DOI:** 10.34172/ijhpm.2022.7580

**Published:** 2022-12-17

**Authors:** Martin Powell, Russell Mannion

**Affiliations:** Health Services Management Centre, University of Birmingham, Birmingham, UK

**Keywords:** Health Policy, Public Policy, Health Policy Processes, Modelling

## Abstract

A range of conceptual models for understanding the policy process have been applied to the health policy process, largely in particular sub areas or policy domains such as public health. However, these contributions appear to offer different rationales and present different frameworks for understanding the policy process. This Editorial critically examines articles that explore the health policy process with models from wider public policy and from health policy. It can be seen that very few of the wider models have been applied in studies of the health policy process. Conversely, some models feature in studies of the health policy process, but not in the wider policy process literature, which suggests that literature on the health policy process is semi-detached from the wider policy process literature. There seem to be two very different future research directions: focusing on ‘home grown’ models, or taking greater account of the wider policy process literature. Does ‘one size fit all’ or is it ‘horses for courses’?

## Introduction

 A range of conceptual models have been developed for describing, understanding and predicting the health policy process.^1^ there have been a number of articles arguing that understanding the health policy process is important, which tend to be discussion of particular sub areas or policy domains such as public health,^2^ health promotion,^3,4^ tobacco control,^5,6^ obesity,^7^ social determinants of health (SDH),^1,8^ and geographical areas such as low- and middle-income countries (LMICs)^9^ ‘Developing Countries’^10^ or sub-Saharan Africa.^11^ However, these contributions appear to offer different rationales and present different frameworks for understanding the policy process. As long ago as 1994, the pioneering work of Walt and Gilson^12^ drew attention to the importance of policy analysis, pointing out that its application to ‘developing countries’ particularly their health sector had been limited. They pointed to the importance of multi-disciplinary policy analysis, asking ‘what can be learned from other disciplines.’ They argued that much health policy wrongly focuses attention on the *content *of reform, and neglects the *actors, processes *and *context. *This led to setting out their ‘model for health policy analysis,’ inspired by political economy approaches, depicted by a triangle with content, context and process at the three apexes, with actors in the middle, which has become known as the ‘health policy triangle.’ Since then, their Editorial critically examines reviews of the policy process in healthcare, and suggests future directions for research on this topic.

## Examining the Policy Process

 John^13^ pointed out researchers use a bewildering array of labels to try to explain and understand policy-making, and there is little agreement on what constitutes a ‘model of the policy process.’ It could be argued that there are two possible approaches: an ‘inductive’ approach which searches for models in relevant public policy journals, and a ‘deductive’ approach that is based on models appearing in well-known texts. We have chosen the second route. However, a large number of texts, some in multiple editions, examine the policy process. For reasons of space, we have chosen perhaps the best-known text is Weible and Sabatier,^14^ which is the fourth edition of ‘Theories of the Policy Process,’ first edited by Paul Sabatier in 1999. The different editions have featured a slightly different range of models. For example, the first two editions included the ‘stages heuristic’ or ‘policy cycle’ or ‘policy stages’ model. Moreover, it presented five criteria for inclusion: a focus on developing scientific theory of policy processes; the presence of an active research community; a comparative research approach; an effort toward making research as public as possible; and continual growth in knowledge about policy processes. Moreover, one of the leading articles on health policy analysis point to this text for an overview of frameworks and theories of the public policy process.^15^ It focused on seven theoretical approaches to policy process research: Advocacy Coalition Framework (ACF), Multiple Streams Framework (MSF), Punctuated Equilibrium Theory (PET), Policy Feedback Theory (PFT), Narrative Policy Framework (NPF), Institutional Analysis and Development Framework (IADF), and Innovation and Diffusion Model (IDM) (compared in chapter 8), which have been used in many different policy areas. However, a number of approaches seem to have emerged from the health policy literature. Both are outlined in Box 1.

**Box 1. Theories of the Policy Process**

**ACF:** policy-making is characterised by the interaction of advocacy coalitions within a policy subsystem. Here, advocacy coalitions comprise of a range of actors from a variety of institutions who share a common set of policy beliefs.

**IDM: **consider what explains the adoption of new policies and how they diffuse across states and other jurisdictions.

**IADF: **aimed at guiding inquiry of how institutions, including public policies, shape human interactions as well as how they are designed and perform.

**MSF:** examines how three independent streams (problem, policy, political) come together to open a policy window, sometimes influenced by a policy entrepreneur.

**NPF: **explores how narratives influence public opinion, how these narratives are structured, and how they reflect policy beliefs.

**PFT:** addresses policy formulation and change, focusing on questions of policy design and dynamics.

**PET:** explores how and why political systems, generally characterized by stability and incrementalism, occasionally produce large scale departures from the past.

** Health Policy Process Sources**

**3-iF:** policy change results from three key elements, or explanatory variables: institutions (processes, context); interests (actors, power) and ideas (content, evidence, values), known as the ‘3Is.’

**Areas of conflict (AoC):** Examines how, why, when do changes occur in public policies and institutions? (agenda-setting, policy formulation, policy implementation). It focuses on factors that influence elites, such as environmental context; circumstances of agenda-setting; and characteristics of the policy.

**GT:** focuses on the activities of actors or groups.

**HAPT: **assumes that an understanding of policy should be informed by an analysis of policy context, content, process and actors, with actors at the centre of the triangle.

**MIT:** depicts whether the policy’s decision making has been dominated by perspectives of either top-down, bottom-up or the synthesis of both of them

**Networks (N):** policy-making takes place in networks consisting of various actors, with the two main ‘network’ models being the ACF and ‘policy and issue networks’ (with the main features of membership, integration, resources, and power).

**PTF: **see HAPT above.

**SDH Framework:** The Commission on the Social Determinants of Health developed SDH Framework for illustrating the two types of health determinants (structural, intermediary determinants) that affect health equity.

**SMT: **arguing that disenchanted people will join social movements to mobilize resources and political opportunity so policy is changed to serve their interests.

**Stages (St):** the policy process follows clearly distinguishable steps from problem definition, through alternative speciﬁcation, to resource allocation and implementation (also known as the ‘stages heuristic’ or the ‘textbook model’).

--------------------

Abbreviations: ACF, Advocacy Coalition Framework; IDM, Innovation and Diffusion Model; IADF, Institutional Analysis and Development Framework; MSF, Multiple Streams Framework; NPF, Narrative Policy Framework; PFT, Policy Feedback Theory; PET, Punctuated Equilibrium Theory; 3i-F, 3-i Framework; AoC, areas of conflict; GT, Group Theory; HPAT, health analysis policy triangle; MIT, Multiple Implementation Theory; PTF, Policy Triangle Framework; SDH, social determinants of health; SMT, Social Movements Theory.

## Studies of Models of the Health Policy Process

 There have been many articles that have reviewed models of the health policy process (see Table). We focus on reviews which emerged from a ‘Google Scholar’ search of ‘models’ AND ‘policy’ and ‘health.’ One of the first contributions was Exworthy^1^ who explored three models of streams (ie, MSF); networks (including ACF), and stages to consider how they contribute to improved understandings of how the SDH policy process.

**Table:** Policy Process and Health Policy Process Models

**Policy Process Models (Weible and Sabatier 2017)**								**Health Policy Process Models**									
	**MSF**	**PET**	**PFT**	**ACF**	**NPF**	**IAD**	**IDM**										
Exworthy (2008)^1^	Y			Y				N	St								
Breton and de Leeuw (2011)^3^	Y			Y						SMT							
Moloughney (2012)^2^	Y	Y	-	Y		Y											
Cullerton et al (2015)^15^	Y			Y													
Clarke et al (2016)^7^	Y			Y			Y				HPAT						
Qudsiah et al (2017)^10^	Y										HPAT	GT	MIT	3-iF	SDHF		
Arabloo et al (2018)^6^	Y	Y		Y		Y					HPAT						
Baum et al (2018)^8^	Y	Y		Y				N	St								
Browne et al (2019)^4^	Y	Y	Y	Y	Y	Y		N			HPAT						
Jones et al (2021)^11^	Y			Y		Y			St		HPAT					AoC	‘Bricolage’

Abbreviations: Y, yes; ACF, Advocacy Coalition Framework; IDM, Innovation and Diffusion Model; IAD, Institutional Analysis and Development; MSF, Multiple Streams Framework; NPF, Narrative Policy Framework; PFT, Policy Feedback Theory; PET, Punctuated Equilibrium Theory; 3i-F, 3-i Framework; AoC, areas of conflict; GT, Group Theory; HPAT, health analysis policy triangle; MIT, Multiple Implementation Theory; SMT, Social Movements Theory; N, Networs; St, Stages.

 Gilson and Raphaely^9^ provided the first critical review of literature analysing the health policy processes of LMICs of 164 articles. They found that less than 40% of the articles demonstrated awareness of the wider field of policy analysis by referring to relevant concepts or theories, and so they categorized them crudely by policy stage such as agenda setting, policy formulation and policy implementation. The most commonly used overarching framework is Walt and Gilson.^12^ However, there was a stronger focus on the earlier stages of policy development (eg, MSF) than on implementation (eg, top down/bottom up theory; street-level bureaucracy). They examine methodological and analytical rigour, but judgements are difficult as the level of detail provided in articles is often fairly limited. For example, around one-third of articles provide very limited details on their data sources, or data collection and analysis methods.

 Breton and de Leeuw^3^ aimed to reﬂect on the state of policy research in health promotion and to examine how rigorously theories are applied. They reviewed literature in 11 peer-reviewed journals between 1986 and 2006, finding that out of the 119 eligible articles, 39 did apply to some degree a theoretical framework, of which 21 (18%) referred to a theoretical framework from political science. They found ‘scant references to theoretical frameworks of the policy process’ (p. 87), with only two papers report on results guided by the ACF and by the MSF, with three papers based on the Social Movement Theory. The majority of the remaining ones used theories of the political science in a more superficial way and in some cases only as a token of acknowledgement of the existence of a policy process, while a few articles reporting on policy processes applied theories from outside the realm of political science. They concluded that ‘this review demonstrates that policy research in health promotion is still largely an a-theoretical enterprise’ (p. 82).

 Moloughney^2^ carried out a literature review to determine the use of policy frameworks to understand public health-related public policy processes. From an initial 376 records, he found 21 policy analysis case studies including a wide range of policy issues across the spectrum of public health. The majority (14) used MSF, four applied ACF, two used PEF, one used IADF. While no study used the Stages Heuristic in isolation, some studies incorporated it into their preliminary descriptive analysis. He stated that the methodological quality of studies was only moderate, with the average quality score of 10.8 out of a maximum of 24, with a range of 4-19.

 Cullerton et al^16^ aimed to determine whether a policy process theory had been used to examine the public health nutrition policy process. They found 63 papers from an initial figure of 1932 sources, with use of policy process theory in 9 (14 %) of the reviewed papers: MST (5), ACF (3) and MST and ACF (1), although five papers referred to policy process theory in their introduction or discussion but it was unclear whether they utilised theory in analysing their data.

 Clarke et al^7^ carried out a systematic review and meta-synthesis of the application of theories of the policy process to obesity prevention, which identified 17 studies of obesity prevention policy underpinned by political science theories. They identified 19 theories of the policy process (their Table 1), but only some six theories appeared in studies of obesity prevention policy processes (their Table 3): MSF, ACF, Institutional Theory, NPF, IDM, and Health Analysis Policy Triangle (HPAT). They noted that three of the theories identified (ACF, MST and PET) have been described as ‘synthesis’ theories, in that they explicitly draw on multiple constructs from more than one other political science theories, and have been often described in the literature as superior to other (non-synthesis) theories in providing an understanding of both policy stasis and change. They stated that many of the included studies were methodologically limited, in regard to rigour and trustworthiness. They concluded that their review demonstrated that there has been limited application of political science theories of the policy process to the study of obesity prevention, with most studies having been in the USA or UK context, and that the limited application of political science theories indicates a need for future theoretically based research into the complexity of policy-making and multiple influences on obesity prevention policy processes.

 Qudsiah et al^10^ used a scoping systematic review to find 18 articles six for each methodological approaches: Theories such as Group Theory, Multiple Implementation Theory, and MSF are among the commonest theories used in policy analysis. In translating framework approaches into health policy analysis, HPAT, 3-i Framework and SDH framework are also widely used.

 Arabloo et al^6^ set out a systematic review aiming to review the application of policy analysis frameworks in the field of tobacco control. They found 17 studies, including MSF (6), PAT (3), ACF (3), and PET (3). However, they considered that the quality of some of the papers was low, with some not explaining the methodology well, not mentioning the source of information, the number of key informants or the way of choosing them. Moreover, a number of studies used the existing frameworks incompletely and superficially.

 Baum et al^8^ stated that it is rare for theoretical insights to be applied to develop understanding about how to increase support for, and the effectiveness of, policy work to address the SDH. They reported on theories and concepts applied during the Workshop to understand why SDH occupies a marginal position on the policy agenda, which included framing the ‘problem’ and establishing the policy agenda (including MSF, ACF, networks, and PET); policy formulation (including MSF); policy implementation; and monitoring and evaluation of policy. However, during the Workshop MSF was drawn on most frequently, and several references were made to traditional conceptions of policy stages and cycles.

 Browne et al^4^ aimed to describe and critique different approaches to policy analysis to provide direction for undertaking policy analysis in the ﬁeld of health promotion. They outlined three broad orientations to policy analysis: traditional approaches, interpretive approaches, and mainstream approaches which focus on the interaction of policy actors in policymaking. Their ‘mainstream’ approaches (their Tables 2 and 3) seemed to consist of: MST, ACF, PET, Institutional Analysis and Development, and policy network analysis.

 Jones et al^11^ explored which theories and conceptual frameworks have been used in research on policy processes of health financing policy in sub-Saharan Africa. They conducted a scoping review of literature published in English and French between 2000 and 2017, resulting in 23 papers, from political science, economics and health policy. The most used frameworks were MST Grindle and Thomas’s^17^ arenas of conflict (26%) and HAPT (30%). However, over a third (35%) papers adopted a ‘bricolage’ approach combining theories and frameworks.

## Discussion and Conclusion

 Our examination of reviews of the policy process in health has broadly endorsed earlier conclusions and raised a new issue. First, it agrees with earlier studies that noted problems of little detail on research design and methodology, the limited use of relevant theory to underpin analysis and the paucity of attempts to provide an explicit, explanatory focus.^9,15^ However, the main finding was that very few of the ‘standard’ approaches from public policy (as provided by our Weible and Sabatier template^14^) have been examined in studies of the health policy process, with only MSF and ACF featuring in all studies, while PET and IAD in a low number of studies. Conversely, some approaches, notably HPAT, feature in studies of the health policy process, but not in the wider policy process literature. Moran^18^ argued that the literature on health-care policy is often semi-detached from the wider literature on the welfare state. This study suggests that literature on the health policy process is similarly semi-detached from the wider policy process literature. There appears to be a heavy focus on agenda setting, with relatively little on implementation. It is perhaps ironic that the huge influence of an article that stressed the importance of looking beyond health policy^12^ has perhaps contributed to its continuing insularity, with much stress on a the health policy triangle, a policy analysis framework specifically for health, although its relevance extends beyond this sector.^15^ Moreover, commentators have noted concerns about the methodological rigour of some of the literature.^2,6,9^

 There seem to be two very different future directions. On the one hand, it might be suggested that studies of the health policy process should focus on ‘home grown’ models, which have been designed to fit with the particular contingencies and features of healthcare, and perhaps also to particular geographical contexts such as LMICs. On the other hand, it might be suggested that studies of the health policy process must become less semi-detached, and take much greater account of the wider policy process literature,^13,14^ with a presumption that studies should be based on models used in the wider literature, unless a good case can be made for drawing on models that tend to be used only in the health policy process literature. This is because it is important to connect with the vibrant research work on these models in the wider public policy literature. This seems to fit with the ‘theoretical’ suggestions of Walt et al^15^ of more critical application of existing frameworks and theories of the public policy process to guide and inform health policy inquiry, and even more broadly, greater use of social science theories (eg, street level bureaucrats) that come from outside of policy studies to inform health policy analysis. Moreover, in our view, the wider policy process models tend to be conceptually stronger than the health policy process models, which tend to point to lists of important variables (eg, interests, institutions, and ideas) rather than examining how these are mutually connected (eg, agents such as policy entrepreneurs in the MST literature), and in which contexts (say) interests are more important than ideas. Put another way, it is likely that the public process theory models would score more highly in ‘tests’ or a ‘policy shootout’^13,14^ compared to the health policy process models.

 There may be a trade-off between policy models tailored to the health sector which are capable of taking into account particular contingencies and features of healthcare as against more generic public policy models which may appear more robust across sectors, but are less sensitive to particular issues facing healthcare contexts? Does ‘one size fit all’ or is it ‘horses for courses?

## Ethical issues

 Not applicable.

## Competing interests

 Authors declare that they have no competing interests.

## Authors’ contributions

 MP and RM contributed equally to the writing of this article.
